# Prediction of twin pregnancy preeclampsia based on clinical risk factors, early pregnancy serum markers, and uterine artery pulsatility index

**DOI:** 10.12669/pjms.37.7.5041

**Published:** 2021

**Authors:** Yan Lu, Zhongying Ding, Wenwen Li, Lina Mei, Linglong Shen, Huaying Shan

**Affiliations:** 1 Dr. Yan Lu, Department of Obstetrics and Gynecology, Huzhou Maternity and Child Health Care Hospital, Huzhou 313000, Zhejiang Province, P.R. China; 2 Dr. Zhongying Ding, Department of Obstetrics and Gynecology, Huzhou Maternity and Child Health Care Hospital, Huzhou 313000, Zhejiang Province, P.R. China; 3 Dr. Wenwen Li, Department of Obstetrics and Gynecology, Huzhou Maternity and Child Health Care Hospital, Huzhou 313000, Zhejiang Province, P.R. China; 4 Dr. Lina Mei, Department of Obstetrics and Gynecology, Huzhou Maternity and Child Health Care Hospital, Huzhou 313000, Zhejiang Province, P.R. China; 5 Dr. Linglong Shen, Department of Obstetrics and Gynecology, Huzhou Maternity and Child Health Care Hospital, Huzhou 313000, Zhejiang Province, P.R. China; 6 Dr. Huaying Shan, Department of Obstetrics and Gynecology, Huzhou Maternity and Child Health Care Hospital, Huzhou 313000, Zhejiang Province, P.R. China

**Keywords:** Twin pregnancy, Preeclampsia, Prediction

## Abstract

**Objectives:**

To investigate whether a combination of clinical risk factors, early pregnancy serum markers, and uterine artery pulsatility index (UTPI) can be used to predict twin preeclampsia (PE).

**Methods:**

This case control study included women with twin pregnancies who had undergone obstetrics treatments and gave birth at the Huzhou Maternity and Child Health Care Hospital from October 2018 to November 2020. Patients with PE comprised study group, and patients without PE comprised control group based on selection criteria and a 1:1 ratio. Statistical analysis was performed using clinical risk factors, early pregnancy serum markers, and UTPIs, and the area under the receiver operating curve (AUC. Sensitivity, and the specificity of different combinations of these variables were calculated to predict PE in women with twin pregnancy.

**Results:**

Logistic regression analysis revealed four independent predictors for the onset of PE during twin pregnancies: first delivery (OR, 7.51; P=0.045), conception method (OR, 7.11; P=0.036), β-HCG level (per SD OR, 2.73; P=0.026), and UTPI (OR, 0.17; P=0.043). First-delivery and IVF pregnancy methods both lead to a 7-fold increase in the PE risk during twin pregnancies. Every one sigma (standard deviation) increase in the β-HCG level led to a 2.73-fold increase in the PE risk. Every UTPI increment by 1.0 reduces the risk of PE by 83%. The prediction efficiencies were based on an AUC of 0.837, a sensitivity of 69%, and a specificity of 92% for the clinical risk factors; an AUC of 0.800, a sensitivity of 81%, and specificity of 78% for the β-HCG level, and an AUC of 0.814, a sensitivity of 88%, and a specificity of 65% for the UTPI. AUC was 0.928, sensitivity 85%, and a specificity 88% after applying the three types of indicators together for prediction.

**Conclusions:**

By combining early pregnancy serum markers (β-HCG), and UTPI, the predictive value for PE during twin pregnancy is improved together with its sensitivity and specificity.

## INTRODUCTION

Preeclampsia (PE) is a serious late pregnancy complication, characterized by pregnancy-induced hypertension, usually accompanied by abnormal cardiovascular, endocrine, and nervous system changes. PE is associated with adverse maternal and perinatal outcomes, such as severe hypertension, heart failure, premature birth, placental abruption, neonatal ischemic hypoxic encephalopathy, and is the second leading cause of maternal and early neonatal mortality in developing countries.^1^ The incidences of twin pregnancies and their complications have increased with the development of assisted reproductive technologies. The risk of PE in women with twin pregnancy is 2-3 times that of women with singleton pregnancy.^2^ Therefore, recent guidelines recommend multi-factorial combined assessment methods to improve the early prediction efficiency for PE.^3^,^4^ However, most studies on PE prediction have focused on singleton pregnancies and few reports exist on multiple pregnancy PE prediction. We investigated clinical risk factors, serum markers, and uterine artery pulsatility index (UTPI) during early twin pregnancy as predictors of twin pregnancy PE.

## METHODS

Data from women with twin pregnancy with or without PE who gave birth at the Huzhou Maternity and Child Health Care Hospital from October 2018 to November 2020 were collected.

### Inclusion Criteria:

Twin pregnancies; primiparous or multiparous women; age range of 18-40 years.

### Exclusion criteria

Three or more multiple-pregnancies; abnormal uterus; selective fetal reduction; missed prenatal appointments during pregnancy; fetuses with chromosomal or structural abnormalities; unexplained miscarriage or stillbirth; treatment with anticoagulants (aspirin or heparin).

PE diagnostic criteria were based on the ACOG guidelines, and collected according to the case control study principles.^3^ Data from 26 women with twin pregnancies and diagnosed with PE (study group) were matched 1:1 to 26 women without PE (control group). The delivery times for each pair were within two weeks from each other. All women underwent first trimester ultrasound examinations to determine the fetal head and hip diameters, gestational age, fetal neck zona pellucida thickness, and twin chorionicity. All data (including the patient’s prenatal examination and hospitalization information) were retrieved from the Zhejiang Provincial Maternity and Child Health System and the electronic medical record system of the hospital, and was used to identify early pregnancy clinical risk factors for PE. The collected information included age, pre-pregnancy body mass index (BMI), conception method (natural conception, artificial insemination, IVF), family history (mother or sister with a history of PE), birth history (pregnancy, parity), and smoking history.

For Uterine artery pulsatility index (UTPI), women from the case study and control groups were examined with ultrasound during the second trimester (ranging from 11+6 to 13+6 weeks) by, experienced personnel. Ultrasound machines used included the GE ultrasound Voluson 730, E8, E6 expert, and the GE LOGIQ E9 ultrasound with 5-9 MHz transvaginal and 3.5 MHz abdominal probes. Women were in a supine position with controlled breathing intensity. After locating the signal from the uterine artery blood flow, the technician manually measured more than three cardiac cycle spectrograms. UTPI value was obtained using an automated computation.

For serum marker detection, 8 ml peripheral blood samples were drawn from all women in the study during the first trimester (between 6 and 14 weeks). Blood supernatants were stored at -20°C, and gradually thawed at 4°C one day prior to the test. All tests were performed after the diagnosis by personnel with no prior knowledge of the clinical information of the participants. level of β-human chorionic gonadotropin (β-HCG) was measured with a PerkinElmer’s 1235 time-resolved fluorescence immunoassay and its corresponding hAFP/Free Hcgβ Dual kit, according to manufacturer’s instruction.

A nested case-control study method was used to analyze the association between the clinical risk factors, serum markers, and UTPI values to PE. We drew receiver operating characteristic curves (ROCs) to calculate areas under the curve (AUCs), sensitivity, and specificity, and to evaluate the PE predicting values during twin pregnancies.

All procedures performed in studies involving human participants were in accordance with the ethical standards of the ethics committee of the Huzhou Maternity and Child Health Care Hospital (2019-R-005, 2020-R-007) . All patients signed the consent.

### Statistical Analysis

Statistical analyses was done using the SPSS20.0 software. Normality and homogeneity of variance tests was applied on measurement data; normally distributed and homogeneous data were represented by means + SDs, and group comparisons were done using *t*-tests. Non-normally distributed data were represented by means and 95% confidence intervals (CIs), and the Mann-Whitney U-test was used for comparisons between groups. We expressed comparisons of counts with rates, and analyzed them using the x^2^ or Fisher’s exact tests. We performed multivariate logistic regression analysis, drew ROC curves, and calculated areas under the curve (AUC), sensitivities, and specificities. A P<0.05 indicates a statistically significant difference.

## RESULTS

A total of 155 women with twin pregnancy from the first trimester to delivery in our hospital were evaluated for the study. Two women terminated their pregnancies before the 24 weeks of gestation due to fetal developmental abnormalities, and four dropped out of the study due to missing follow-ups. Eventually 26 women developed PE, for an incidence rate of PE during twin pregnancy of 17%. Univariate analysis indicated that the patients with PE had higher average age, higher rate of IVF conception, and earlier gestational weeks of delivery than the women in the control group, with statistically significant differences (P<0.05). BMI values, pregnancy times, parity, chorionic properties, family PE history, and smoking history were all similar between groups (P>0.05), Table-I.

**Table I:** Comparison of the general conditions of the two groups of pregnant women.

*Variable*	*Twin pe (n=26)*	*Control group (n=26)*	*X*2/t**	*P value*
Age (years)			10.833	0.001
20-34	15(57.7)	25 (96.2)		
35-40	11 (42.3)	1 (3.8)		
BMI(kg/m2)			0.977	0.738
<18.5	4 (15.4)	5 (19.2)		
<25	14 (53.8)	16(61.5)		
≥25	8 (30.7)	5 (19.2)		
Pregnancy times			0.316	0.854
1	11 (42.3)	13 (50.0)		
2	9 (34.6)	8(30.8)		
≥3	6(23.1)	5(19.2)		
parity			2.564	0.109
0	22(84.6)	17(65.4)		
≥1	4(15.4)	9(34.6)		
Conception method			8.571	0.014
Natural pregnancy	9(34.62)	19(73.1)		
Ovulation	2(7.7)	2(7.7)		
IVF	15(57.7)	5(19.2)		
Fetal chorionic properties			2.185	0.139
Single cashmere twins	6(23.1)	11(42.3)		
Double cashmere twins	20(76.9)	15(57.7)		
Family PE history			0.354	0.5
Yes	2(7.7)	1(3.8)		
No	24(92.3)	25(96.2)		
Smoking			1.02	0.5
Yes	0(0)	1(3.8)		
No	26(100)	25(96.2)		
Gestational week of delivery (weeks)	35.8±1.6	36.8±1.8	17.245	0.001

**Note:** Age, BMI, times of pregnancy, parity, method of conception, nature of fetal chorion, family PE history, smoking cases are expressed as percentages (%), and gestational week of delivery is expressed as x±sd.

**Fig.1:**
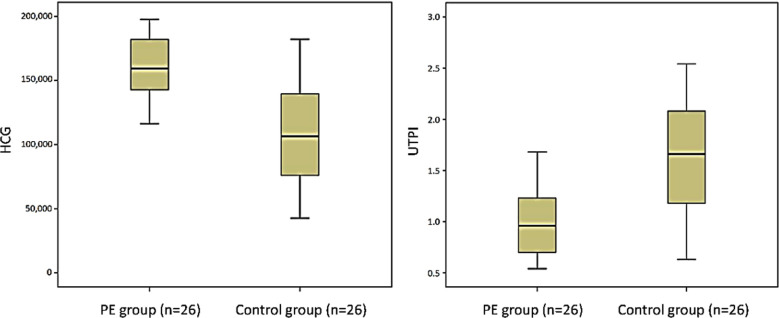
HCG and uterine artery pulsatility index

Mean β-HCG level of women with PE was significantly higher than that of the control group, and the mean UTPI level was lower than that of the control group (P<0.05). Fig.1 and Table-II.

**Table II:** Comparison of serum markers and UTPI between the two groups.

*Group*	*n*	*β-HCG (mIU/ml)*	*UTPI*
PE group	26	161251.77±22533.35	0.98±0.32
Control group	26	104822.85±38857.63	1.63±0.60
t		6.406	4.695
P value		<0.001	<0.001

**Note:** Data is expressed as x±sd.

Taking the occurrence of PE as the dependent variable (non-PE twins=0, PE twins=1), age, parity, serum markers (β-HCG), and UTPI were used as independent variables. We assigned values to the independent variables (age, 20- 34 years=1, 35-39 years=2; parity, 0 times=1, ≥1 times=2). The logistic multivariate analysis results showed that the first delivery [(odds ratio (OR), 7.51; 95% CI, 1.04 to 54.18; P=0.045], conception method (OR, 7.11; 95% CI, 1.14 to 44.36; P=0.036), and β-HCG level per 1SD (OR, 2.73; 95% CI, 1.13 to 6.60; P=0.026) were independent risk factors for the onset of PE in twin pregnancies. Primiparous women who have used IVF conception methods had a 7-fold increased risk of PE during twin pregnancy. For every β-HCG level standard deviation elevation, the risk of PE increased 2.73 times (P <0.05). For UTPI (OR, 0.17; 95% CI, 0.031 to 0.944; P=0.043), and every 1.0 increase reduced the risk of PE by 83% (P <0.05). Table-III.

**Table III:** Multivariate Logistic regression analysis of the two groups.

*Parameter*	*Or*	*95%ci*	**P* value*
Parity	7.51	1.04-54.18	0.045
Conception method	7.11	1.14-44.36	0.036
β-HCG(mIU/ml)(per 1 SD)	2.73	1.13-6.60	0.026
UTPI	0.17	0.031-0.944	0.043
Age	2.537	0.156-41.292	0.513

**Fig.2:**
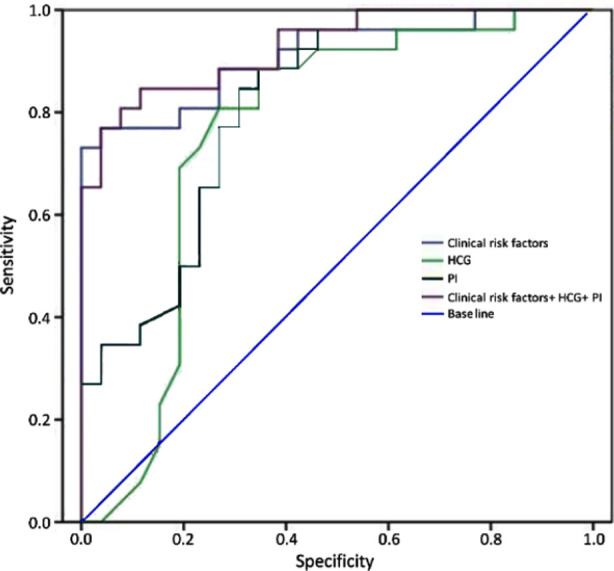
ROC of each variable to predict preeclampsia of twin individually and jointly.

The prediction accuracy of using clinical risk factors (parity + conception method), biomarkers, and the UTPI together to screen women with twin pregnancy for PE is shown in the ROC curve in Fig.2; the area under the ROC curve, sensitivity, and specificity are shown in Table-IV. By adding biomarkers and UTPI, the screening performance of the clinical risk factors for twin PE was improved. When the false positive rate (FPR) is 5%, using a combination of clinical risk factors, serum markers, and UTPI, increases the prediction accuracy for PE during twin pregnancies based on an AUC of 0.928, sensitivity of 85%, and specificity of 88%.

**Table IV:** The value of each factor for independent or joint PE prediction during twin pregnancies.

*Parameter*	*Sensitivity*	*Specificity*	*AUC*	*95%CI*	*P value*
Clinical risk factors (parity + method of conception)	69	92	0.837	0.726+0.947	<0.001
β-HCG	81	78	0.8	0.677-0.924	<0.001
UTPI	88	65	0.814	0.697-0.930	<0.001
Clinical risk factors +β-HCG+UTPI	85	88	0.928	0.860-0.995	<0.001

## DISCUSSION

This study examined the ability of various clinical risk factors and biomarkers to predict PE in women with twin pregnancy. It found four independent predictors for PE onset during twin pregnancy: first delivery, conception method, β-HCG level, and UTPI. Predictive power, as well as sensitivity and specificity, improved when multiple factors were combined.

The onset of PE is earlier for women with twin pregnancy, and is associated with higher maternal and perinatal mortality.^5^,^6^ Therefore, early prediction and detection of PE are essential to improve outcomes. Current guidelines recommend continuous low-dose (75-162mg/d) aspirin to people at high risk of PE between 16 to 36 weeks of gestation, to prevent PE and improve maternal-fetal pregnancy outcomes.^7^-^9^ However, due to the side effects of the drug, not every woman with a twin pregnancy should take aspirin, and usually compliance is low.^10^ Developing early PE prediction methods for twin pregnancies will allow timely identification of women with twin pregnancies who would benefit from the treatment to avoid non-selective aspirin administration.

Recent studies suggest a two-stage model of PE pathogenesis: placental superficial implantation followed by placental oxidative stress and inflammation.^8^ The incidence of PE in twin pregnancies may also be associated with maternal factors, such as high body mass index (BMI) and high blood coagulation tendency.^11^,^12^ A study by Taguchi et al.^13^ on 742 women with twin pregnancies found that first-time delivery, high BMI, family history of hypertension, and history of hypertension during a previous pregnancy were all significant risk factors for PE with ORs at 1.77 (95% CI, 1.21 to 2.61), 1.35 (95% CI, 1.08 to 1.70), 1.50 (95% CI, 1.02 to 2.17), and 8.85 (95% CI, 2.70 to 29.0), respectively. Benko et al.^14^ used maternal factor information from 2219 women with twin pregnancy as a training data set to build a PE prediction model, tested data from 2999 women with twin pregnancy, and reported the outcome of early onset PE prediction. The AUCs for all PE cases were 0.670 (95% CI, 0.593-0.747) and 0.677 (95% CI, 0.594 to 0.760), the predicted AUCs for all PE cases were 0.656 (95% CI, 0.615 to 0.697) and 0.644 (95% CI, 0.606 to 0.682).

Studies show that the frequency of PE during twin pregnancies is two to three times higher than during singleton pregnancies, and the incidental rate ranges from 13% to 26%.^15^,^16^ Consistently with these reports, we found that 17% of women with twin pregnancy in our cohort developed PE. The results of our multivariate logistic analysis indicated that first-time delivery pregnancy and IVF conception methods were significantly associated with PE during twin pregnancies. Both first-time delivery (OR, 7.51; 95% CI, 1.04 to 54.18; P=0.045) and conception method (OR, 7.11; 95% CI, 1.14 to 44.36; P=0.036) were associated with a 7-fold increase in PE risk. The false positive rate (FPR) was 5%, and the detection rate of PE during twin pregnancies was 69%.

PE has been linked to vascular and endothelial abnormalities. During normal pregnancies, thickness of the inner diameter of the uterine artery increases to meet the changing demands in placental blood supply, and the blood flow switches from its normal state of high resistance and low discharge to a pregnant state of low resistance and high discharge. As the pregnancy progresses, the uterine artery blood flow resistance drops significantly, and the UTPI gradually decreases. In patients with PE, number of remodeling uterine spiral arteries is reduced, due to the incomplete erosion by trophoblast cells. This results in relatively high vascular resistance, while the uterine artery blood flow is characterized by high resistance and low discharge^17^ accompanied by significantly elevated UTPI. Uterine artery blood flow parameters have become a common method to predict the risk of PE during singleton pregnancies.^18^ The consensus states that in both singleton and twin pregnancies, the uterine artery blood flow resistance is decreased with increase in gestational age, and that UTPI during twin pregnancies is consistently lower than that of singleton pregnancies.^19^,^20^ However, there are only few inconclusive studies on the association between UTPI levels in women with twin pregnancy and PE. Lack of consensus may be due to UTPI measurements done at different gestational weeks.^21^,^22^ We found that the mean UTPI in women with twin pregnancy and PE was lower than in women without PE. UTPI in the twin PE group (OR, 0.17; 95% CI, 0.031 to 0.944; P=0.043) suggests that every UTPI increase by one reduces the risk of PE by 83%. When the FPR is 5%, the AUC for PE is 0.814 with UTPI alone, and when the sensitivity is 88%, the specificity is 65%. For women with twin pregnancy and PE, abnormal blood supply of one placenta may cause a compensatory increase in the blood supply to the other placenta.^23^ Due to the limited sample size in our study, we did not analyze the UTPI characteristics of women with identical twins and fraternal twins and PE. The mechanisms leading to abnormal UTPIs in women with twin pregnancy and PE need to be studied.

Many studies focused on serum biomarkersin general and β-HCG in particular, as predictors and prognostic factors of PE.^24^, ^25^

β-HCG is mostly synthesized by placental trophoblastic cells, which are important indicators of placental function.^26^ However, few studies have focused on the changes in serum β-HCG in women with twin pregnancies and PE. Metz et al.^27^ found that the serum β-HCG level during the first trimester of twin pregnancies is higher than that during singleton pregnancies, and that the serum β-HCG level in people with twin pregnancies that go on to develop PE is significantly higher than that of women without PE (P= 0.004). Iskender et al.^28^ confirmed that the serum β-hCG level in women with twin pregnancies and PE was higher than in women with normal twin pregnancies, and that a high β-hCG level was associated with adverse pregnancy outcomes such as fetal growth restriction. Our results show that everyone standard deviation from the normal β-HCG levels increases risk of PE 2.73-fold (OR,2.73; 95% CI, 1.13 to 6.60; P=0.026). When the FPR is 5%, the AUC in women with twin pregnancy and PE alone is 0.800, and when the sensitivity is 81%, the specificity is 78%.

### Study Limitations

The study has two main limitations. The first is a small sample size, which prevented us from investigating certain relationships and associations, such as UTPI characteristics of women with PE who bore identical twins vs. those who bore fraternal twins. The second is that the study population was relatively homogenous, since all patient data was collected through a single regional health facility. Additional investigations on larger and more heterogeneous study populations will need to be performed to further evaluate the predictive power of the highlighted parameters and biomarkers.

## CONCLUSION

In summary, this study demonstrated that combining early pregnancy serum markers (β-HCG) and UTPI markedly improves their sensitivity, specificity, and overall predictive value for PE during twin pregnancy. Future studies with large sample sizes and long follow-ups are needed to establish a PE predictive model for twin pregnancies that has a low false positive rate and a high detection rate. The chosen model should improve the early diagnostic efficiency for PE during twin pregnancies to provide a reliable diagnostic basis for effective treatments and interventions.

### Authors’ contributions:

**YL:** Conceived and designed the study.

**ZD, WL, LM, LS, HS & YL:** Collected the data and performed the analysis.

**YL:** Prepared the manuscript.

**YL:** Was responsible for the integrity of the study.

**All** authors have read and approved the final manuscript.
